# Dental Hints Department

**Published:** 1907-07

**Authors:** B. H. Teague

**Affiliations:** Aiken, S. C.


### ...DENTAL HINTS DEPARTMENT...

CONDUCTED BY B. H. TEAGUE, D. D. S., AIKEN, S. C., TO WHOM ALL CONTRIBUTIONS TO THIS DEPARTMENT SHOULD BE SENT

The human body, owing to the salts which it contains, conducts nearly twenty times better than water, when the skin is well moistened.—Dr. H. L. Ambler, Dentists Mag.

Stovaine.—Many reports seem to indicate that stovaine is rapidly replacing cocaine as a local anesthetic, safer and as effective or more so.—Jour. Clin. Med.

Cocain retards the action of the arsenic, requiring a much longer period to produce the result, but permits of pulp destruction without the slightest disturbance if the application is properly made.—Dentist's Mag.

We know that Anesthesia is a pathological and not a physiological condition. From this we must observe that ABSOLUTE SAFETY in the use of any anesthetic does not exist; for, where there is a pathological condition there is danger.—Dr. S. Strauh, Dentists Mag.

Mouth Antiseptics.—The different permanganates, especially the permanganate of zinc are not only effectual as mouth antiseptics, but pleasant and far superior to the sodium and potassium salts. One tablet (one-tenth of a grain) should be dissolved in half a tumbler of water.—Wyatt Wingrane, Lancet.

Don't shun plate work. If you are going to locate in a small town your ability will be judged largely by your plate work, so the sooner you decide to cultivate this side of the business, the better it will be for your pocketbook.—Dr. D. D. Campbell, Ozart Mo.

Little Helps.—A magnet is a very useful article at the chair. A broach drops on the floor, or some part of your midget separator, or several burs. The magnet will find these when you can't—time is saved and temperature kept at the normal!—Geo. Zederbaum, Dental Register.

To Clean Plates.—To remove calcareous deposits from rubber plates, drop them in a solution of muriatic acid and water; one part of acid to ten of water. Result is immediate.—Dr. G. G. Brown, Western.

Annealing Platinum.—We find that many will bring platinum to a pretty good red heat and believe it is annealed; but practically nothing is done in the way of annealing until platinum is heated to a white incandescence, and until then you have softened it very little.—C. L. Hungerford, Western Dental Journal.

Do not use Varnish as a Cavity Lining.—I have long ago discarded all the varnishes—as in my experience they invariably oxidized and discolored far more than they ever decolored—I have used oxyphosphates instead.—Dental Cosmos.

Solution to Remove Stains of Silver Nitrate and Other Salts Sensitive Dentin.—Ammonium Chloride, 1 part; Mercury Bichloride, 1 part; water, 8 parts. Apply and wash off with soap and water.—W. H. Tweedle, Review.

Light for the Dentist.—Dr. Bing, of Paris, operates in a dark room, the only light admitted being that conveyed by means of a movable tube to the face of the patient. This is an ideal arrangement so far as the eyesight of the dentist is concerned, illuminating the mouth with the least expenditure of energy by the occular apparatus.—Dr. Casey Wood, Dental Review.

The dawning of progressive and humanitarian dentistry is at hand, you can no more stop the tide of progress than you can turn back the sun in the Universe nor with your poluted fingers reach out to tarnish it.—Dr. J. K. Conray, Era.

In Orthodontia.—The sets of models of the teeth (upper and lower) should always be made, one set to keep for comparison and one upon which to construct appliances. The importance of having a sufficient number of good, properly articulated models cannot be too strongly emphasized.—Dentist's Mag.

Acute Suppuration.—Applications of carbolic acid—camphor mixtures are more efficacious than dressings with any of the ordinary agents employed in cases of acute suppuration. Pure carbolic acid 1 ounce, alcohol 2½ drachms, camphor, 2 ounces.—C. Ehrlich, Semaine Medicale.

The Quill Toothpick.—In my practice I preach the gospel of the quill toothpick. Its use makes a great difference in the condition of the teeth; on account of its sharp edges it will scale off the soft deposits where the wooden one will not. —J. S. Hall, Dental Register.

To Remove Tin From Vulcanite.—Small particles of tin adhering to vulcanite plates can easily be removed by mixing mercury with enough alloy so it will not flow and rubbing it over the plate under fingers.—C. W. Siefkill, Dental Brief.

Tightening Screw Connections.—For tightening screw connections, dissolve powdered shellac in 10 per cent. ammonia and paint the mass over the screw threads after they have been thoroughly cleaned; then screw the fitting home. The joint will be impervious to hot or cold water.—Popular Magazine.

Care of Nickel Plated Parts.—One of the best methods known for keeping bright the nickel work about the office is to wet a rag with a solution of hyposulphite of soda and wipe the article with it, drying with a soft towel and then rubbing with a piece of chamois.—Bulletin.

Grinding Teeth.—In grinding up a full upper and lower the process is practically the same, only the surfaces are reversed. In the upper the buccal cusps should be at a sharper angle, while the lingual cusp should be somewhat rounded. The lower is just the opposite—lingual sharp and buccal rounded.—Dr. W. D. James, Tracy, Minn.

The Horns of the Dilemma.—According to Miller, the bacteria increase more rapidly in alkaline than in acid solution; in an acid mouth the acid will cause tooth decay; in an alkaline mouth the bacteria will grow more rapidly, finally generating an excess of acid that will attack the teeth.—Joseph Head, Dental Cosmos.

The Berry Crown Metal.—

| | |
|---|---|
| Bismuth | 16 parts |
| Tin | 16 parts |
| Lead | 10 parts |
| Antimony | 4 parts |

—Dentists Mag.

To Abort Boils.—It is possible to prevent suppuration in a small proportion of forming boils. As soon as the local inflammation is noted, the following is to be applied:

| | |
|---|---|
| Fluid extract of ergot | 2.0(dr. ½) |
| Oxide of zinc | 8.0(drs. 2) |
| Phenol | 0.5(grs. 8) |
| Lanolin | 65.0(ozs. 2) |

This is to be spread on gauze or absorbent cotton to the size of a silver dollar, applied over the boil, and held by adhesive plaster. It should be replaced by belladonna ointment in twelve hours, but may be repeated next day if the boil is still red and painful. [Calcium sulphide internally. —Ed.], Jour. Clin. Med.

Arsenic.—The writer was very much gratified recently when at a recent meeting of one of our prominent dental societies this question was brought before the body and a vote was taken to ascertain how many of the men were using arsenous acid, and it was found that almost the entire membership was using it to a greater or less extent. This was all the more gratifying because within a year or two had a similar vote been taken few indeed would have been those who dared express themselves as advocates of that drug.—Editorial Dentists Magazine.

The Care of the Eyes.—In the morning it is unwise to exert the eyes immediately after rising, and it is advisable during the dark early hours to dress by the aid of as little artificial light as possible in order that the eyes may become gradually accustomed to their function. In very bright daylight during the summer, window shades should not be opened or raised suddenly, because, although few persons realize it, the change from darkness to brilliant light is likely to strain the strongest eyes.—Exchange.

Formaldehyde.—The great value of formic aldehyde lies in its great power of penetrating tissues, sterilizing as it goes. In teeth which have been treated, and in which every attempt to seal them up is followed by pain and tenderness, a 5 per cent. solution used as a dressing on a wisp of cotton and left in for a week will remedy all that, and the tooth may be filled with the assurance that all will be well.—B. J. T. Bennette, Dental Record.

One Method of Handling Sensitive Dentin.—Use carbolic acid as strong as it can be used, and by that means, solidifying the albumenoids contained in the dental tubulli. Then use chloride of zinc in crystals, and allow it to deliquesce. Eighteen or twenty years of this kind of practice has, notwithstanding the statements of some of the most learned professors, not caused the death of a single pulp, and permits the cutting of sensitive dentine with little or no pain.—Dr. E. O. Bogan, Dental Review.

A recent bulletin of the Kansas State Board of Health bears on its front page the following motto: "The best natural disinfectant, sunshine; the best germ disinfectant, formaldehyde; the best physical disinfectant, soap; the best moral disinfectant, publicity." The first is dispensed in daytime; the second, in bottles; the third, hard or soft, in cakes or cans respectively; the fourth, in either articulate speech or daily paper, preferably the latter because of its inexpensiveness and far-reaching results.—Off. and Lab.

Extracting Live Pulps.—When one has extracted the pulp down to its finest termination curling as it dies on the Donaldson broach, one is sometimes surprised on pushing up a cotton-wound broach to dry the canal, to get an expression of acute pain that makes one wonder whether by any chance, a bit of pulp remains. Persisting in the effort to dry the canal, the pain is gone, and one may vigorously prod without causing any sensation. I did not realize how acutely painful a little moisture could make a tooth until I had the experience in a tooth of my own.—C. P. Briggs, Amer. Jour.

A Reliable Cocaine Preparation.—My experience having proven that none of the cocaine solutions on the market or elsewhere are reliable, as cocaine rapidly deteriorates when in solution, I have used with excellent results a one to two per cent. solution of phenol sodique, adding to a syringeful, a 1-6 gr. tablet of cocaine. This I have found more effective than any solutions that have stood for an indefinite length of time.—Dr. A. D. Barker, Summary.

Frequently we see cases where one or both of the upper lateral incisors are missing and there is a wide space between the centrals. A space is also left where the lateral incisors should be, but no indication of these teeth in the jaw anywhere, and a radiograph reveals no sign of them. In observing these cases you will often note that the cuspid teeth are not long nor do they have well developed crowns; always short crowned, or as it seems, they do not extrude from the gum as far as the ordinary tooth does, and the central incisors are usually not perfectly formed teeth.—Dr. Hoff, Register.

Solidified Formaldehyde.—While not a new medicament, has not, to our knowledge been reported within the past year. This is produced by heating the aqueous solution in a sandbath with sufficient heat to form a gas, which instead of evaporating, becomes polymerized as soon as the concentration exceeds 40 per cent., increasing in density until solid, which when placed in the pulp chamber coming in contact with the heat and moisture of the tooth liberates formaldehyde gas which has almost entirely lost its caustic properties. Having been sealed in the cavity it finds its way to all portions of the root canal producing thorough sterilization.—Items of Interest.

Spence-Metal.—Spence-metal is much used by the European dentists for dies and counter dies for gold plates. The chief component parts of Spence-metal are iron and sulphur; its fusing point is lower than that of boiling water and it hardens quite rapidly. It must be melted over a very slow fire, otherwise it may catch fire with a very suffocating odor of sulphur. Stir with a wooden stick until it presents a very thick, bubbling liquid, then extinguish the fire immediately and leave undisturbed until crystals begin to form at the sides of the vessel—the opportune moment for casting the die. Pour quickly to the edges of the flask because of rapid hardening. It can be poured into a plaster-of-paris impression, first lubricating the impression with oil or vaselin.—Dr. M. Bank, Western Dental Journal.

Tender Mucous Membrane.—Too much tobacco smoking will make the mucous membrane tender and irritable, and so also will the bad habit of wearing dentures at night.

It is, when recognized, the best thing to advise moderation in the first of these habits, and to refrain from the second, and after the cases are removed at night to gargle the mouth with some astringent such as tannin or myrrh, and repeat it in the morning before the insertion of the cases. This will strengthen and brace up the gums to withstand, with comfort, the extra pressure they are to be subjected to.—Harry Rose, Dentist's Mag.

Cocaine.—That cocaine can and should be used in many, if not in most cases, is possibly true, but that there are many cases where arsenous acid should be used, to the exclusion of all else, is an indisputable fact. But that cocaine is used in hundreds of cases where it is contra-indicated is also an indisputable fact. In taking up a discussion of question, it is done from the humanitarian standpoint. There are many cases where it is utterly impossible to use cocaine without inflicting considerable pain in so doing, and there are many cases in which it is an utter impossibility to remove all the nerve tissue from the canals of the teeth by this method. It is the writer's belief that in all teeth, having small and and tortuous canals, it it absolutely necessary to use arsenous acid in the destruction of the pulp tissue if the most satisfactory results are to be accomplished.—Dentist's Mag.

"Soft" and "Hard" Teeth.—It may now be regarded as firmly established that dental caries does not occur because of any faults in the calcification of the teeth further than that pits, grooves and accidental physical imperfections give greater opportunity for the cause of caries to act. Also that there is no such thing as soft teeth and hard teeth in the sense that these phrases have heretofore been used. Neither susceptibility to decay nor immunity from decay are in any wise dependent upon variations in chemical composition of the tissues of the teeth, but must be dependent upon the oral secretions and what they may contain that enables the fermentative process to develop substances that may act upon them, and localize that action.—G. V. Black, Dental Review.

Treatment after Scaling.—After scaling place the rubber dam over the teeth to be retained, including one more at each end, then if the cuspids are fairly firm, begin with one and weave a fine gold or gold and silver wire around the incisors and tie to the other cuspid trying to draw the irregular teeth into line; then cover this wire with a solution of celluloid dissolved in acetone the consistency of cream, working this around and between the teeth over the wire into the shape of bands. After a couple of hours the patient may be dismissed with a coating of sandrach to protect the celluloid and the next day with a sharp knife, stones and disks, the celluloid may be trimmed and polished. This makes a good temporary retainer, one which does not separate the teeth nor cause much pain. For the celluloid cement use celluloid in thin sheets obtained at any art store; add acetone same as you would mix shellac varnish.—Dr. H. H. Harlan, Items.

Use of the Blue Light.—The contrivance is a simple 16-candle power blue electric light globe arranged in a funnel shaped tin shield which, at its mouth, is about four inches in diameter; this is extended about four inches and has at its end a round blue glass and convex lens. The round blue glass is used to disseminate the blue rays so that the patient may not know the simplicity of the apparatus, and I attribute no especial virtue to the lens. Dr. Watkins claims that in cases of acute abscess, impacted third molars and their associated lesions he has used the blue light with great effect, relieving the pain and causing the disappearance of the inflammatory condition usually attending this form of disease.—Dr. J. C. Watkins, Items of Interest.

Teeth Forms as Guides.—It has occurred to me quite recently that a set of vulcanite forms, such as those used in technic work, representing a full upper and lower arranged in characteristic imitation of the various temperaments indicated, would prove a valuable guide, not only to the student and young practitioner, but even to the man of experience, in the placing of teeth upon the articulator. A great majority of those in practice today are spending such a large portion of their time in the restoration and repair of the natural teeth, and their thoughts are so concentrated along these lines, that they have neither time nor disposition to devote to this phase of prosthetic work.—Dr. F. J. Moyer, Summary.

An Effective Ligature.—In cases where the clamp is objectionable for the retention of the rubber dam, and where the ordinary floss is not sufficiently bulky to prevent the rubber from drawing over it, a most admirable method of using the ligature is to first pass the floss through two pieces of rubber tubing, allowing one piece on the buccal and one on the lingual side of the tooth. This is much preferable to stringing beads on the ligature or using other agents, as already suggested. The tubing should be the smallest size sold ot the rubber stores, the kind used for slipping over the bows of glasses where they rest on the ears. To insure against leakage, drop a little sandarac varnish between the tubing and the enamel on buccal and lingual sides.—Dr. E. M. S. Fernandez, Review.

The Step Anchorage and Its Indications.—We are seldom if ever justified in placing a filling in the approximal surface of a bicuspid or molar where the occluding tooth is in position and the occlusion is normal, without securing it by a step anchorage cut as a right angle to the pulpal wall into the occlusal fissure. If this is done, and the step is cut sufficiently broad and deep making that portion slightly broader at the end farthest from the cavity, there will seldom be any movement of the filling to the cavity, but care must be exercised in preparing the step to make it sufficiently deep and broad to insure sufficient filling material to prevent its stretching or breaking by the force of occlusion in the course of mastication.—J. F. Wallace, Dental Era.

Flexible Rubber Border.—In The Summary was described a method of putting a flexible rubber border on a plate. I think my method simpler, viz.: See to it that the trial-bite plate is trimmed so that the plate will not infringe on any muscles. Wax, invest, pack the regular vulcanite rubber and close the flask tightly in hot water; cool and separate. Trim away with sharp knife all excess rubber that has forced out and scrape a very shallow groove all around the margin on the model. Then place a narrow strip of palate or flexible rubber all around the rim of the regular vulcanite, to correspond to the groove; close flask and vulcanize in usual manner. If properly prepared previous to investing, it will not be necessary to trim the edge.—Dr. A. D. Barber, Summary.

A Tooth Saving Age.—I will say that this is a progressive age, an age of advancement and progress in all lines of work, skill and thought, and we as dentists should advance with the times—this is a tooth saving age; at least it ought to be, from the number of dental colleges and the courses of study and the number of graduates that go out each year from them. The day for the physician, horse doctor, blacksmith and barber pulling teeth has gone. The dentist of today and the future will be expected to possess the necessary skill to save teeth and not pull them. The treating and saving of abscessed teeth is the most difficult and trying ordeal the dentist has to contend with. Nevertheless, we owe it to the public, to ourselves, and to the profession.—Summary.

The Trend of Medicine.—The trend of medicine is toward prophylaxis. The stomatologist in treatment of interstitial gingivitis has the advantage of the general practitioner since he is thereby able to prevent the tendency to more grave disorders. Many times have I told my patients after urinalysis, that they were suffering with autointoxication which required treatment. They would later return with the statement from the family physician that they were in perfect health. Had the family physician recognized the tendency to grave disease portrayed in the mouth, his diagnosis would have been far different. The patient with interstitial gingivitis due to autointoxication is sick so far as the stomatologist is concerned, although able to come to the office and attend to his duties. He is not sick in the eyes of the family physician since the symptoms have not markedly manifested themselves constitutionally.—Dr. E. S. Taloot.

Objection to Chloroform.—The objections to chloroform have reference to its circulatory effects chiefly. While experimental evidence show that chloroform kills the healthy mammal by paralyzing respiration—and cases are seen where, respiratory failure occurring under this drug, the patient is promptly resuscitated by artificial respiration—yet its great danger lies in the fact that it greatly depresses the heart, and its free or prolonged administration may cause fatty degeneration of that organ. The irritability of the heart muscles is also reduced, so that its response may be deficient.

With chloroform, therefore, when respiration stops we have to reckon not only with a paralyzed respiration, but with a depressed heart as well. The odds may be too great for the organ to recover itself, hence the frequent fatalities from this anesthetic.—Register.

A METHOD OF PARALLELING BRIDGE ABUTMENTS WITHOUT DEVITALIZATION.—Where one or both of the abutment teeth incline toward each other, the teeth are prepared for gold crowns in the usual manner.

A gold cap with parallel sides is fitted to the rear abutment, which, when in position on the tooth, will incline in the same direction as the tooth.

A section of gold plate is now soldered to the cap, so as to fill the space between the front edge of the grinding surface of the cap and the gum margin extending a little in front of a line parallel to the opposite abutment.

This section of gold plate extends to the sides of the cap so as to cover about one-half of the buccal and lingual sides.

The cap and appendage is placed in position and a gold crown is made to telescope over the cap. The remainder of bridge is made in usual way.—H. PHILLIPS, Summary.

HOW TO LIVE.—The most genial crier of "largesse!" Dr. D. K. Pearsone, of Chicago, whose substantial deeds in behalf of education are held in grateful rememberance in Colorado, has announced that he is going to live to be 100 years old. The doctor is now past 80, and the chief material which he intends to use in building the superstructure of the remaining 20 years is sunshine.

The sun bath that is to be his fountain of youth is not the sunshine that rules without, but the sunshine that comes from a contented spirit. Just read a few lines of his long life rules:

"Most men dig their graves with their teeth."

"No pies or cakes, no pains or aches."

"Live like a farmer and you'll live like a prince."

"Don't get angry and don't get excited; every time you fret you lose a minute of life."

"If you catch cold lose your quinine and eat an onion."

"Give away money; it's exhilerating and tends to longevity."

"The idea of giving when one's alive will become epidemic when men discover what fun it is."—WILLIS BARNES, The Dieltetic and Hygienic Gazette.

RELIEF OF PAIN IN BLIND ABSCESS.—Instantaneous and often permanent, relief from pain following infection from a pulpless tooth may be obtained by the use of equal parts absolute alcohol and water. Whether the pain is caused by an upper or lower tooth the treatment is equally effective.

The preparation can best be used with what is known as a watch case atomizer. Fill the atomizer with the solution, then, having the patient recline the head a little, place the nozzle of the atomizer into the nostril on the side where the troubln is, spray the solution back into the nostril twice and the pain will be relieved immediately. In some cases there will be a slight amount of gagging and the solution will come out through the mouth, but this discomfort is only temporary.

If the pain should return within fifteen minutes a stronger solution, say 75 per cent. alcohol and 25 per cent. water, may be used and the treatment repeated, but usually the 50 per cent, solution will be sufficient and will permit you to open up the tooth and give the patient relief.—DR. J. E. KEEFE, Items of Interest.

DIATORIC TEETH IN PLATE WORK.—Thinking that some dentist's on account of the high price of platinum pin teeth, may find it advantagous to use diatoric teeth, I will explain my method of using them to make a finished case with the teeth as firmly attached as they possibly can be, and one that has stood the test. I have been using the Ash & Son's diatoric teeth, as they have small holes running through the body of the teeth which I utilize as follows: Take about 24-gauge iridiumoid wire, pass it through the hole in the tooth, and with stub pliers give it one twist, and cut off with the shears some distance from the twist. Then, with pliers, bend each end of wire forward toward the body of the tooth, forming a small loop of each end. Teeth are then ready to set up, the wire loops taking the place of pins. When these teeth are used in this manner you have a piece of work of which you need have no fear of the pins pulling from the plate, and one that can be finished as artistically as any vulcanite denture.—DR. C. W. MYERS, Summary.

